# Supplementary material for: Single-cell and bulk transcriptional profiling of mouse ovaries reveals novel genes and pathways associated with DNA damage response in oocytes
Source: bioRxiv. 2024 Feb 4:2024.02.02.578648. Preprint. [Version 1] doi: 10.1101/2024.02.02.578648 (PMC10862846; doi:10.1101/2024.02.02.578648)
Supplement: Supplement 1 — Table S1. RRGs identified by bulk RNA-seq. Table S2. g:Profiler analysis of RRGs. Table S3. Gene Set Enrichment Analysis of RRGs. Table S4. Primers used in this study. [file media-1.pdf]

Single-cell and bulk transcriptional profiling of mouse ovaries reveals novel genes and pathways associated with DNA damage response in oocytes.

Monique Mills[1,2], Chihiro Emori[3], Parveen Kumar[1], Zachary Boucher[1], Joshy George[1] and Ewelina Bolcun-Filas[1]*

Affiliations

1 The Jackson Laboratory, 600 Main Street, Bar Harbor, ME 04609, USA.
2 The Graduate School of Biomedical Science and Engineering, University of Maine, Orono, ME 04469, USA
3 Department of Experimental Genome Research, Research Institute for Microbial Diseases, Osaka University, Suita, Osaka 5650871, Japan.
*Corresponding author: Ewelina.Bolcun-Filas@jax.org

Keywords: DNA Damage Response, CHEK2, oocyte, ovary, single-cell transcriptomics

Supplementary Materials:

Supplementary Figures (S1-S7)

Supplementary Tables (S1-S4)

Figure S1.

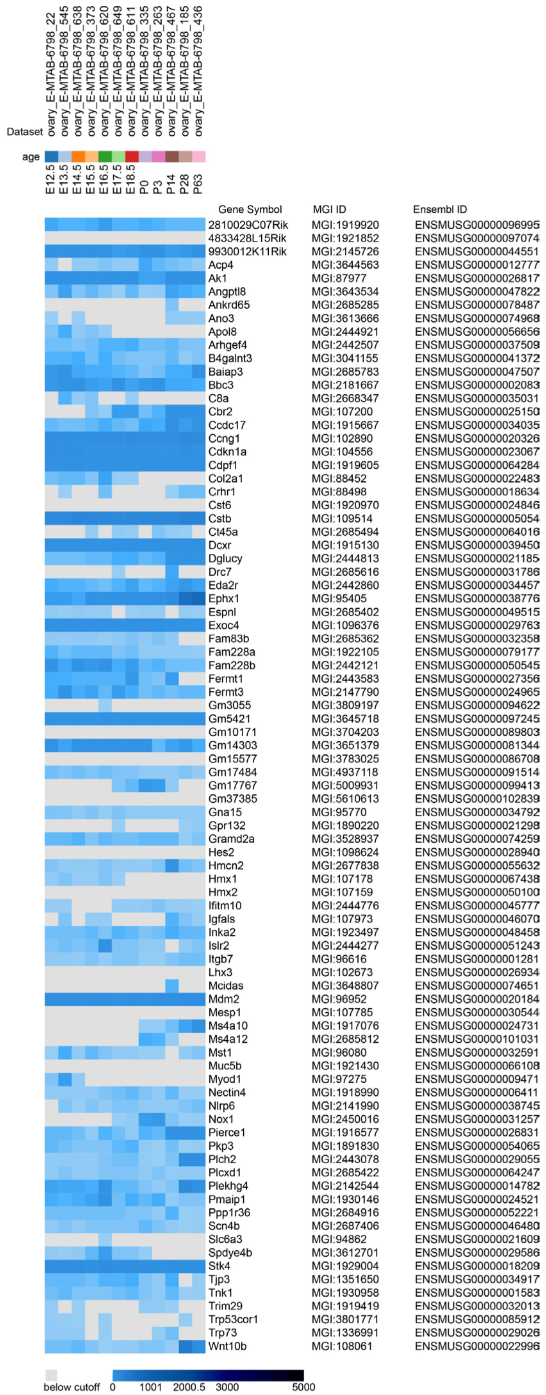

Figure S1. Expression of RRGs identified in the study during ovary development generated using published data and Gene Expression Database (GXD) at Mouse Genome Informatics Portal https://www.informatics.jax.org/expression.shtml

Figure S2.

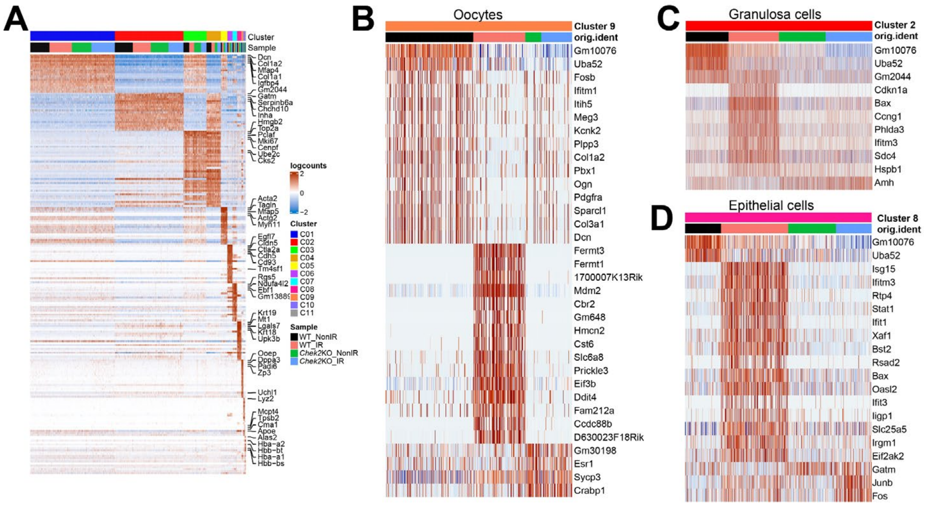

Figure S2. A) Heatmap of marker gene expression from single cells obtained from wildtype and *Chek2-/-* ovaries with and without radiation organized into 11 clusters. B-D) Heatmaps of differentially expressed genes in oocyte, granulosa, and epithelial cell clusters.

Figure S3.

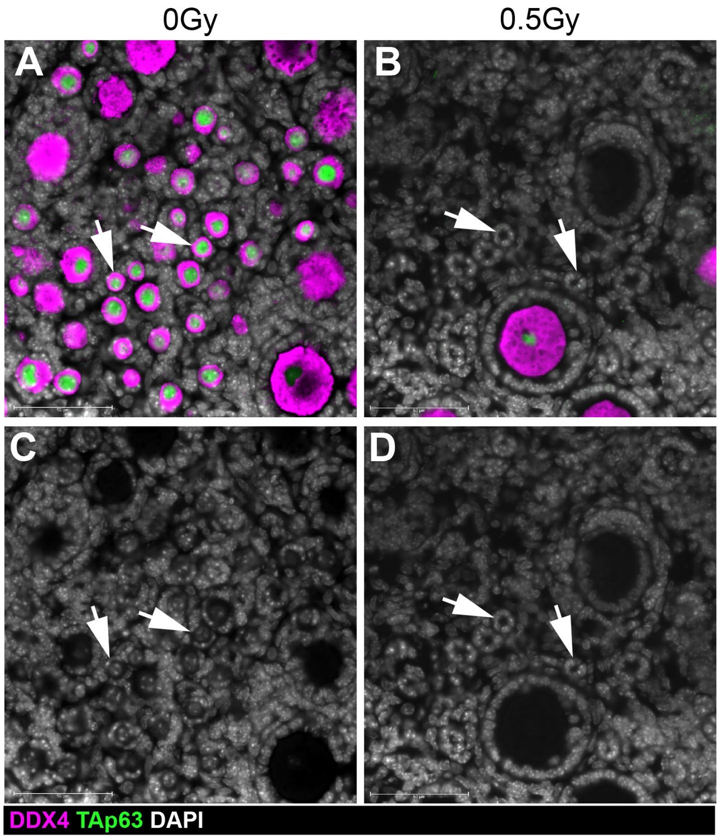

Figure S3. Example image of non-irradiated (A) and irradiated (B) ovaries immunostained with oocyte markers DDX4 and TAp63. C-D) Grayscale image for DAPI channel to visualize nuclei. Arrows indicate primordial follicles with an oocyte inside in non-irradiated (C) and empty follicles with visible granulosa cells but without the oocyte in the irradiated ovary (D).

Figure S4.

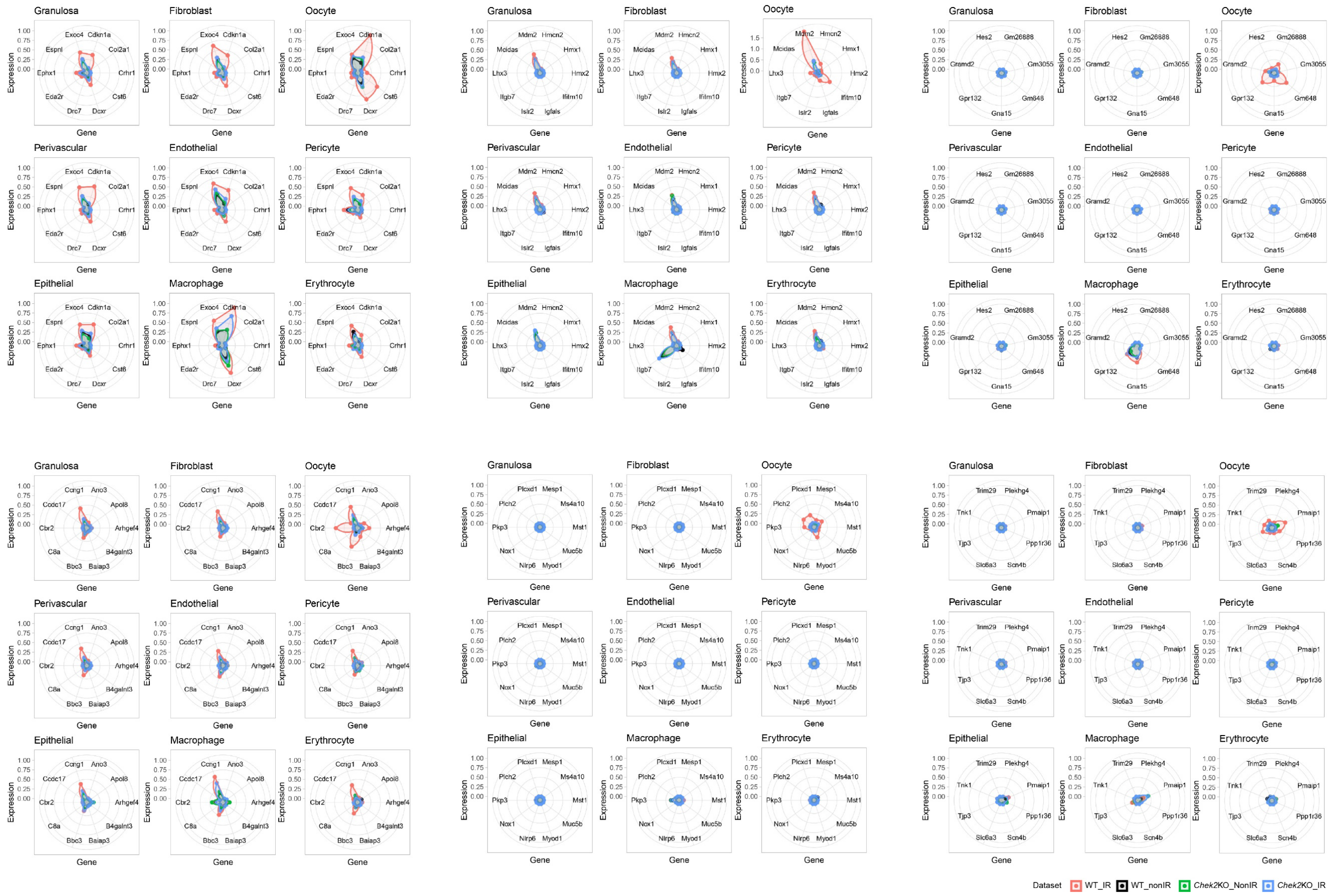

Figure S4. Radar charts showing expression of RRGs in different cell types.

Figure S5.

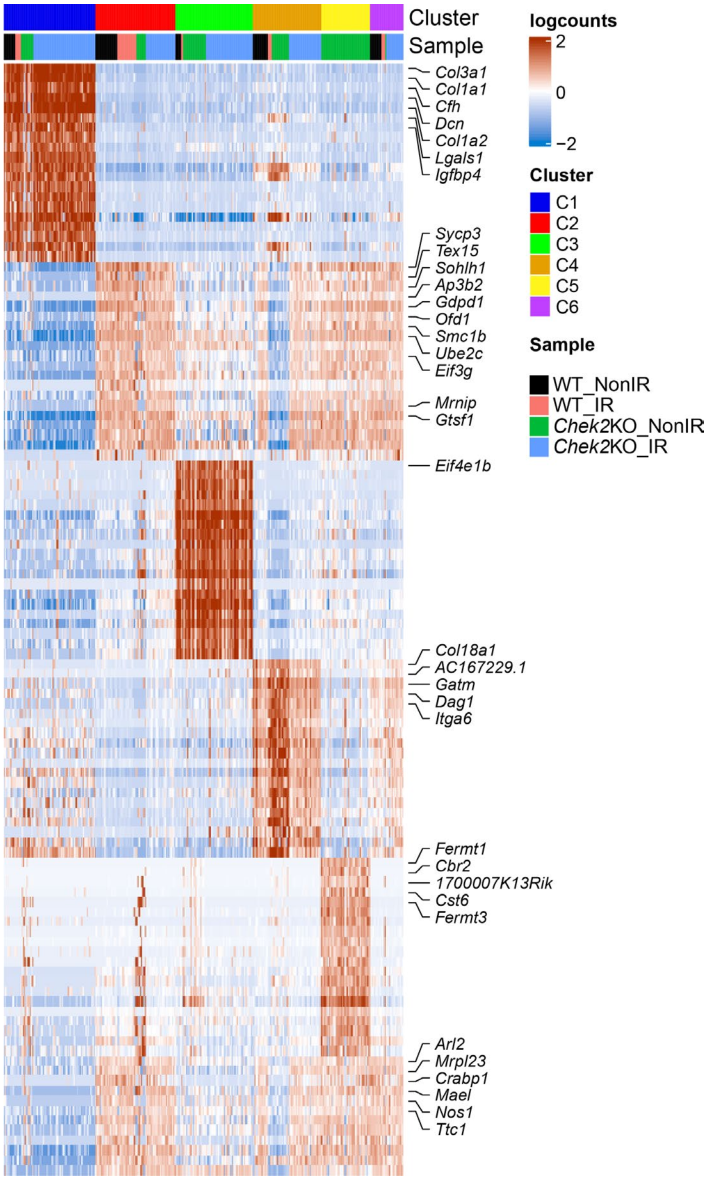

Figure S5. A) Heatmap of marker gene expression for six oocyte subclusters identified by de novo re-clustering.

Figure S6.

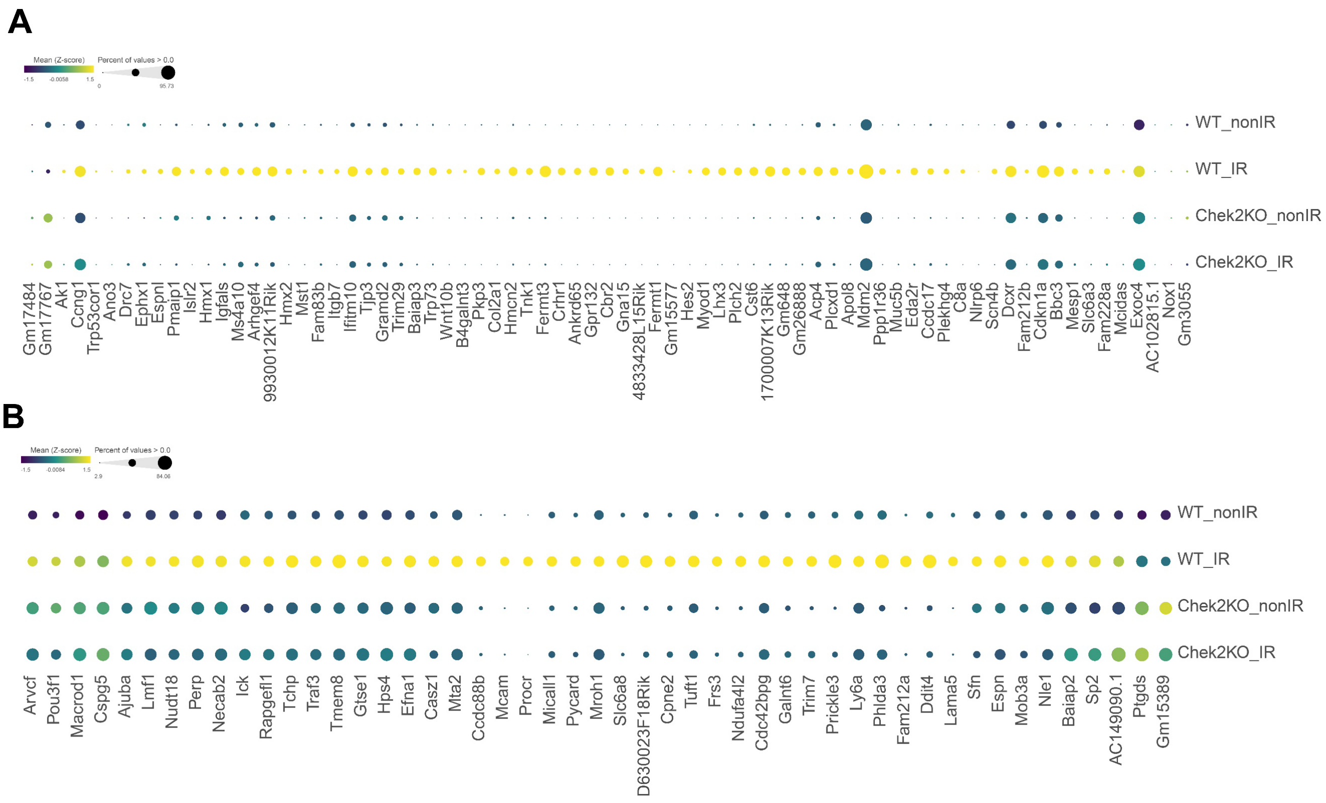

Figure S6. Dot plot showing expression of RRGs from bulk (A) and additional RRGs from subcluster analysis (B) in all oocytes from wildtype and *Chek2-/-* ovaries with and without radiation.

Figure S7.

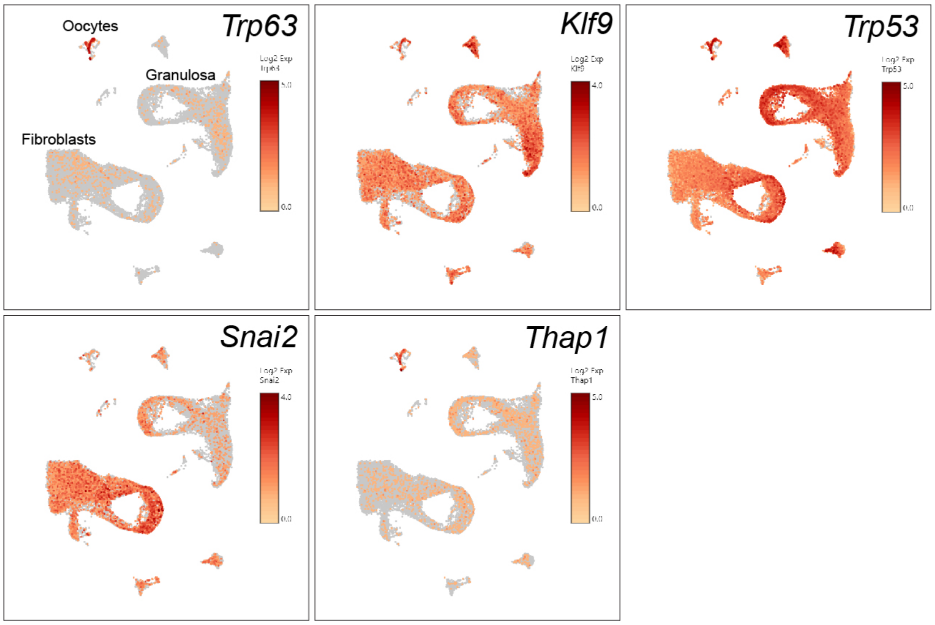

Figure S7. Gene expression levels of transcription factors predicted to regulate RRGs overlaid on UMAP plots with the oocyte, granulosa, and fibroblast clusters indicated.

Supplementary Tables:

Supplementary Table S1. RRGs identified by bulk RNA-seq.

Table S1. Radiation Responsive Genes in the ovary
Part 1. Response in wildtype ovaries.

MGI Gene/ Marker ID	Symbol	LogFC WT	Adj p-value	Feature Type	Gene Ontology terms
**MGI:5610613**	*Gm37385*	22.7	8.77E-05	unclassified gene	
**MGI:3809197**	*Gm3055*	20.6	1.02E-03	protein coding gene	
**MGI:102673**	*Lhx3*	7.8	1.14E-10	protein coding gene	cell differentiation; DNA-binding transcription factor activity, RNA polymerase II-specific
**MGI:1921852**	*4833428L15Rik*	6.9	1.06E-07	lncRNA gene	
**MGI:2685285**	*Ankrd65*	6.7	1.22E-18	protein coding gene	
**MGI:107159**	*Hmx2*	6.5	3.27E-05	protein coding gene	cell differentiation; DNA-binding transcription factor activity, RNA polymerase II-specific
**MGI:2444921**	*Apol8*	5.8	6.63E-08	protein coding gene	chloride channel activity; extracellular space; high-density lipoprotein particle; intracellular membrane-bounded organelle
**No associated gene**	*Gm43301*	5.7	1.22E-04	lncRNA gene	
**MGI:3613666**	*Ano3*	5.6	1.27E-30	protein coding gene	calcium activated phospholipid scrambling; chloride channel activity; chloride transmembrane transport
**MGI:3783025**	*Gm15577*	5.5	5.92E-03	lncRNA gene	
**MGI:107785**	*Mesp1*	5.5	2.99E-03	protein coding gene	DNA-binding transcription activator activity, RNA polymerase II-specific; Notch signaling pathway
**MGI:1098624**	*Hes2*	5.5	6.45E-08	protein coding gene	DNA-binding transcription factor activity, RNA polymerase II-specific; DNA-binding transcription repressor activity, RNA polymerase II-specific
**MGI:2443078**	*Plch2*	5.3	8.57E-51	protein coding gene	calcium ion binding; intracellular signal transduction; phosphatidylinositol-mediated signaling; phospholipase C activity; plasma membrane; signal transduction
**MGI:94862**	*Slc6a3*	5.2	2.50E-11	protein coding gene	cell surface; dopamine transport; integral component of membrane; signaling receptor binding
**MGI:107200**	*Cbr2*	5.1	2.91E-05	protein coding gene	carbonyl reductase (NADPH) activity; glucose metabolic process; mitochondrion; oxidoreductase activity
**MGI:97275**	*Myod1*	4.8	6.15E-07	protein coding gene	cell differentiation; cellular response to estradiol stimulus; cellular response to oxygen levels; cellular response to tumor necrosis factor; DNA-binding transcription activator activity, RNA polymerase II-specific; ubiquitin protein ligase binding
**MGI:88498**	*Crhr1*	4.7	2.07E-09	protein coding gene	adenylate cyclase-activating G protein-coupled receptor; signaling pathway; cell surface receptor signaling pathway; endosome; transmembrane signaling receptor activity; vesicle
**MGI:2685402**	*Espnl*	4.7	2.42E-18	protein coding gene	actin binding; cell projection; stereocilium tip; stereocilium tip
**MGI:2668347**	*C8a*	4.7	1.39E-03	protein coding gene	complement activation; cytolysis; immune response; membrane attack complex
**MGI:2443583**	*Fermt1*	4.5	9.82E-06	protein coding gene	cell adhesion; cell junction; focal adhesion; integrin-mediated signaling pathway; negative regulation of; canonical Wnt signaling pathway; positive regulation of cell adhesion mediated by integrin
**MGI:1890220**	*Gpr132*	4.5	1.42E-15	protein coding gene	G1/S transition of mitotic cell cycle; G protein-coupled receptor signaling pathway; negative regulation of G2/M; transition of mitotic cell cycle; plasma membrane; signal transduction
**MGI:2685616**	*Drc7*	4.4	5.93E-07	protein coding gene	cell differentiation; cell projection; cilium
**MGI:88452**	*Col2a1*	4.3	1.35E-06	protein coding gene	cellular response to BMP stimulus; extracellular region
**MGI:3648807**	*Mcidas*	4.2	2.60E-02	protein coding gene	cell cycle; negative regulation of cell cycle; negative regulation of DNA replication
**MGI:2444776**	*Ifitm10*	4.2	5.11E-37	protein coding gene	integral component of membrane; membrane
**MGI:1916577**	*Pierce1 (1700007K13Rik)*	4.1	1.10E-18	protein coding gene	cellular response to DNA damage stimulus; regulation of gene expression
**MGI:2685494**	*Gm648*	4.1	2.26E-10	protein coding gene	
**MGI:95770**	*Gna15*	4.0	3.05E-05	protein coding gene	adenylate cyclase-modulating G protein-coupled receptor signaling pathway; GTPase activity membrane; phospholipase C-activating G protein-coupled receptor signaling pathway
**MGI:1918990**	*Nectin4 (Pvrl4)*	3.9	3.12E-10	protein coding gene	cell junction
**MGI:1919920**	*2810029C07Rik*	3.9	9.10E-37	lncRNA gene	
**MGI:2444277**	*Islr2*	3.8	2.02E-11	protein coding gene	cell surface; integral component of membrane; protein binding
**MGI:2687406**	*Scn4b*	3.7	2.07E-11	protein coding gene	ion transport; plasma membrane; sodium channel activity
**MGI:2684916**	*Ppp1r36*	3.7	1.11E-09	protein coding gene	negative regulation of phosphatase activity; phosphatase binding
**MGI:3612701**	*Spdye4b (4933411G11Rik)*	3.7	3.15E-03	protein coding gene	protein kinase binding
**MGI:2147790**	*Fermt3*	3.6	4.15E-10	protein coding gene	cell adhesion; cell junction; integrin-mediated signaling pathway
**MGI:3645718**	*Gm5421*	3.6	1.67E-02	pseudogene	
**MGI:2685362**	*Fam83b*	3.3	3.85E-23	protein coding gene	phosphatidylinositol 3-kinase catalytic subunit binding; signal transduction
**MGI:1921430**	*Muc5b*	3.3	4.79E-08	protein coding gene	extracellular matrix; regulation of macrophage activation
**MGI:2444813**	*Dglucy (9030617O03Rik)*	3.3	5.40E-16	protein coding gene	D-glutamate cyclase activity; lyase activity; mitochondrion
**MGI:107178**	*Hmx1*	3.2	2.56E-05	protein coding gene	DNA-binding transcription factor activity, RNA polymerase II-specific
**MGI:3801771**	*Trp53cor1*	3.2	2.03E-04	lncRNA gene	negative regulation of gene expression; ribonucleoprotein complex
**MGI:1920970**	*Cst6*	3.2	4.08E-04	protein coding gene	epidermis development
**MGI:1930958**	*Tnk1*	3.0	7.58E-06	protein coding gene	ATP binding; kinase activity; negative regulation of Ras protein signal transduction; transmembrane receptor protein tyrosine kinase signaling pathway
**MGI:108061**	*Wnt10b*	3.0	1.04E-02	protein coding gene	canonical Wnt signaling pathway; G2/M transition of mitotic cell cycle; negative regulation of transcription by RNA polymerase II; regulation of cell cycle
**MGI:96080**	*Mst1*	3.0	4.59E-04	protein coding gene	extracellular region; negative regulation of epithelial cell apoptotic process; receptor tyrosine kinase binding; regulation of receptor signaling pathway via JAK-STAT serine-type endopeptidase activity
**MGI:1915667**	*Ccdc17*	3.0	7.06E-18	protein coding gene	
**MGI:5477382**	*Gm26888*	2.8	4.60E-09	lncRNA gene	
**MGI:1336991**	*Trp73*	2.8	8.25E-11	protein coding gene	apoptotic process; cell cycle; cell junction; cellular response to DNA damage stimulus; DNA-binding transcription activator activity, RNA polymerase II-specific; intrinsic apoptotic signaling pathway in response to DNA damage by p53 class mediator; MDM2/MDM4 family protein binding; negative regulation of JUN kinase activity; p53 binding; positive regulation of apoptotic process; positive regulation of MAPK cascade
**MGI:1930146**	*Pmaip1*	2.7	1.16E-16	protein coding gene	activation of cysteine-type endopeptidase activity; involved in apoptotic process; positive regulation of apoptotic process; positive regulation of DNA damage response, signal transduction by p53 class mediator; reactive oxygen species metabolic process; regulation of apoptotic process
**MGI:3643534**	*Angptl8 (Gm6484)*	2.6	3.32E-04	protein coding gene	extracellular region; negative regulation of lipoprotein lipase activity
**MGI:1891830**	*Pkp3*	2.6	6.08E-07	protein coding gene	adherens junction; alpha-catenin binding; cell-cell adhesion; cell-cell junction assembly
**MGI:2142544**	*Plekhg4*	2.6	6.96E-03	protein coding gene	activation of GTPase activity
**MGI:1351650**	*Tjp3*	2.5	4.90E-06	protein coding gene	cell-cell adhesion; cell-cell junction; cell surface; regulation of G1/S transition of mitotic cell cycle
**MGI:3644563**	*Acp4 (Acpt)*	2.5	1.09E-07	protein coding gene	acid phosphatase activity; negative regulation of ERBB4 signaling pathway; peptidyl-tyrosine dephosphorylation involved in inactivation of protein kinase activity; protein tyrosine phosphatase activity

**MGI:96616**	*Itgb7*	2.4	2.61E-04	protein coding gene	cell adhesion; cell adhesion mediated by integrin; focal adhesion; integrin-mediated signaling pathway
**MGI:1919419**	*Trim29*	2.4	5.51E-03	protein coding gene	innate immune response; lysosome; negative regulation of transcription by RNA polymerase II; p53 binding
**MGI:1922105**	*Fam228a*	2.4	1.30E-02	protein coding gene	
**MGI:3528937**	*Gramd2*	2.3	5.77E-08	protein coding gene	extrinsic component of cytoplasmic side of plasma membrane; integral component of membrane; phosphatidylinositol binding
**MGI:1917076**	*Ms4a10*	2.1	2.42E-03	protein coding gene	cell surface receptor signaling pathway; integral component of membrane; membrane
**MGI:4937118**	*Gm17484*	2.1	5.54E-05	lncRNA gene	
**MGI:2145726**	*9930012K11Rik*	2.1	1.26E-12	protein coding gene	
**MGI:102890**	*Ccng1*	2.0	5.64E-105	protein coding gene	cell cycle; cell division; cyclin-dependent protein; serine/threonine kinase regulator activity; cytoplasm; mitotic G2 DNA damage checkpoint signaling; negative regulation of apoptotic process
**MGI:87977**	*Ak1*	2.0	1.64E-05	protein coding gene	adenylate kinase activity; regulation of G1/S transition of mitotic cell cycle
**MGI:2685783**	*Baiap3*	2.0	1.95E-05	protein coding gene	calcium ion binding; G protein-coupled receptor signaling pathway; late endosome membrane; regulation of dense core granule exocytosis
**MGI:2685422**	*Plcxd1*	1.9	1.10E-07	protein coding gene	lipid metabolic process
**MGI:2181667**	*Bbc3*	1.9	3.41E-11	protein coding gene	activation of cysteine-type endopeptidase activity; involved in apoptotic process; cellular response to DNA damage stimulus; cellular response to ionizing radiation; intrinsic apoptotic signaling pathway; intrinsic apoptotic signaling pathway by p53 class mediator; PUMA-BCL-xl complex; response to endoplasmic reticulum stress
**MGI:107973**	*Igfals*	1.9	2.92E-02	protein coding gene	cell adhesion; extracellular region
**MGI:2677838**	*Hmcn2*	1.8	1.92E-03	protein coding gene	basement membrane; cell junction; collagen-containing extracellular matrix; response to stimulus
**MGI:1923497**	*Inka2 (Fam212b)*	1.8	2.95E-13	protein coding gene	negative regulation of catalytic activity; protein serine/threonine kinase inhibitor activity
**MGI:2442860**	*Eda2r*	1.8	2.19E-21	protein coding gene	cell differentiation; integral component of membrane; intrinsic apoptotic signaling pathway by p53 class mediator; positive regulation of I-kappaB kinase/NF-kappaB signaling; positive regulation of JNK cascade<br>programmed cell death; tumor necrosis factor-mediated signaling pathway
**MGI:3041155**	*B4galnt3*	1.7	3.26E-03	protein coding gene	acetylgalactosaminyltransferase activity; glycosyltransferase activity; Golgi apparatus
**MGI:95405**	*Ephx1*	1.7	3.26E-03	protein coding gene	integral component of membrane; plasma membrane; response to toxic substance
**MGI:104556**	*Cdkn1a*	1.7	5.54E-05	protein coding gene	cell cycle; cellular response to DNA damage stimulus; cellular response to ionizing radiation; cellular response to UV-B; cyclin-dependent protein serine/threonine kinase inhibitor activity; DNA damage response, signal; transduction by p53 class mediator resulting in cell cycle arrest; mitotic G2 DNA damage checkpoint signaling; PCNA-p21 complex; regulation of cell cycle G1/S phase transition
**MGI:1915130**	*Dcxr*	1.6	2.91E-03	protein coding gene	carbonyl reductase (NADPH) activity; L-xylulose reductase (NADP+) activity; membrane; oxidoreductase activity; positive regulation of reactive oxygen species; metabolic process
**MGI:2442507**	*Arhgef4*	1.5	6.22E-04	protein coding gene	intracellular signal transduction; lamellipodium assembly; protein domain specific binding
**MGI:1096376**	*Exoc4*	1.4	9.61E-06	protein coding gene	cell projection; exocytosis; regulation of protein transport; small GTPase binding; vesicle tethering involved in exocytosis
**MGI:96952**	*Mdm2*	1.3	3.40E-07	protein coding gene	apoptotic process; cellular response to gamma radiation; DNA damage response, signal transduction by p53 class mediator resulting in cell cycle arrest; NEDD8 ligase activity; p53 binding
**MGI:5009931**	*Gm17767*	-2.2	4.93E-03	lncRNA gene	
**MGI:2450016**	*Nox1*	-2.6	7.06E-05	protein coding gene	cell junction; cellular response to hyperoxia; endosome; extracellular matrix organization; NADPH oxidase complex; oxidoreductase activity; positive regulation of JNK cascade; positive regulation of MAPK cascade; positive regulation of oxidative stress-induced intrinsic; apoptotic signaling pathway; small GTPase binding
**MGI:2141990**	*Nlrp6*	-3.1	8.69E-03	protein coding gene	activation of cysteine-type endopeptidase activity; acute inflammatory response; ATP binding; inflammasome complex; necroptotic process; negative regulation of ERK1 and ERK2 cascade; negative regulation of I-kappaB kinase/NF-kappaB signaling; NLRP6 inflammasome complex
**No associated gene**	*AC102815.1*	-15.2	1.96E-02	unclassified gene	
**MGI:3704203**	*Gm10171*	-20.2	1.64E-07	pseudogene	
**MGI:3651379**	*Gm14303*	-22.2	1.71E-04	pseudogene	

Part 2. Response in *Chek2-/-* ovaries.					
**MGI Gene/ Marker ID**	**Symbol**	**LogFC *Chek2-/-***	**Adj p-value**	**Feature Type**	**Gene Ontology terms**
**MGI:5610613**	*Gm37385*	26.4	1.77E-05	unclassified gene	
**MGI:1338893**	*Padi1*	-6.7	0.032901	protein coding gene	calcium ion binding; hydrolase activity; protein-arginine deiminase activity
**MGI:102673**	*Gm43520*	-22.9	1.77E-05	unclassified gene	

Supplementary Table S2. Gene enrichment analysis of RRGs with g:Profiler.

Source	Term Name	Term ID	Adj.pvalue	-log10 adj.pvalue	Genes
**GO:BP**	intrinsic apoptotic signaling pathway by p53 class mediator	GO:0072332	0.001	3.15	*Eda2r, Pmaip1, Bbc3, Trp73, Mdm2, Cdkn1a*
**GO:BP**	signal transduction by p53 class mediator	GO:0072331	0.019	1.71	*Eda2r, Pmaip1, Bbc3, Trp73, Mdm2, Cdkn1a*
**KEGG**	p53 signaling pathway	KEGG:04115	0.000	6.62	*Ccng1, Pmaip1, Bbc3, Trp73, Mdm2, Cdkn1a*
**KEGG**	Platinum drug resistance	KEGG:01524	0.001	3.05	*Pmaip1, Bbc3, Mdm2, Cdkn1a*
**KEGG**	Apoptosis - multiple species	KEGG:04215	0.013	1.88	*Pmaip1, Bbc3*
**KEGG**	Human papillomavirus infection	KEGG:05165	0.024	1.62	*Hes2, Mdm2, Col2a1, Cdkn1a, Itgb7, Wnt10b*
**KEGG**	Colorectal cancer	KEGG:05210	0.036	1.44	*Pmaip1, Bbc3, Cdkn1a*
**WP**	p53 signaling	WP:WP2902	0.000	7.07	*Ccng1, Pmaip1, Bbc3, Trp73, Mdm2, Cdkn1a*
**WP**	Apoptosis	WP:WP1254	0.004	2.41	*Pmaip1, Trp73, Mdm2*
**WP**	Hypoxia-dependent self-renewal of myoblasts	WP:WP5023	0.009	2.04	*Myod1, Cdkn1a*
**MIRNA**	mmu-miR-23a-3p	MIRNA:mmu-miR-23a-3p	0.030	1.52	*Pmaip1, Bbc3*

Supplementary Table S3. Gene Set Enrichment Analysis of RRGs (GSEA).

HALLMARK PATHWAY	ES	NES	NOM p-val	FDR q-val	TOP GENES (HUMAN SYMBOLS)
**P53 PATHWAY**	0.67	2.20	0.00	0.00	*CCNG1, AK1, CLCA2, CDKN1A, DCXR, EPHX1, MDM2, PIDD1, PHLDA3, BTG2, ZMAT3, ZNF365, GLS2, MAPKAPK3, EPHA2, SFN, BAX, SLC19A2, DEF6, DDIT4*
**INTERFERON ALPHA RESPONSE**	0.67	2.05	0.00	0.00	*DHX58, RTP4, TRIM14, IRF9, LGALS3BP, TMEM140, TAP1, EIF2AK2, SAMD9L, PARP9, IRF7, HELZ2, PROCR, DDX60, TRIM21, ISG15, PSMB8, IFITM3, GBP4, HERC6*
**INTERFERON GAMMA RESPONSE**	0.57	1.86	0.00	0.00	*CDKN1A, ITGB7, DHX58, OAS2, RTP4, TRIM14, IRF9, LGALS3BP, TAP1, EIF2AK2, SAMD9L, DDX58, XAF1, STAT1, IRF7, HELZ2, DDX60, CSF2RB, TRIM21, ISG15*
**APOPTOSIS**	0.53	1.70	0.00	0.01	*PMAIP1, GNA15, CDKN1A, BTG2, BAX, TAP1, CD14, GUCY2D, IFITM3, CASP7, MMP2, GADD45A, IER3, CASP1, PLCB2, GSR, FAS, HGF, DPYD, TNFSF10*
**TNFA SIGNALING VIA NFKB**	0.51	1.69	0.00	0.01	*CDKN1A, BTG2, TAP1, EGR2, KYNU, DDX58, EGR1, DRAM1, LIF, F3, DUSP2, PLEK, PTGER4, EFNA1, IFIH1, LAMB3, SOCS3, IL7R, FOS, PLK2*
**INFLAMMATORY RESPONSE**	0.49	1.61	0.00	0.02	*GPR132, GNA15, CDKN1A, BTG2, RTP4, EIF2AK2, IRF7, LIF, RNF144B, BEST1, F3, CD14, ADORA2B, PTGER4, P2RX7, SEMA4D, HRH1, IL7R, ITGB8, CD48*
**IL6 JAK STAT3 SIGNALING**	0.52	1.56	0.01	0.03	*IRF9, STAT1, IL1R2, CSF2RB, CD14, CCR1, SOCS3, CXCL9, DNTT, IL18R1, SOCS1, STAT2, CSF3R, FAS, PIK3R5, EBI3, MAP3K8, ACVR1B, PF4, CD44*
**APICAL JUNCTION**	0.47	1.54	0.00	0.03	*EXOC4, NECTIN4, COL17A1, NFASC, MAP3K20, CLDN4, ACTA1, PPP2R2C, CDH4, PARD6G, CLDN14, LIMA1, GRB7, VCL, BAIAP2, ICAM5, THY1, MAP4K2, CDH3, LAMB3*
**ESTROGEN RESPONSE LATE**	0.47	1.53	0.00	0.03	*PKP3, DCXR, TRIM29, TJP3, RAPGEFL1, OVOL2, SFN, ASS1, CLIC3, ST14, CA2, CACNA2D2, TOB1, SCNN1A, MAPT, PERP, DUSP2, TFAP2C, CELSR2, PGR*
**COMPLEMENT**	0.46	1.52	0.00	0.03	*TMPRSS6, ADRA2B, CDK5R1, CA2, KLK1, MMP15, KYNU, IRF7, F3, PLEK, CASP7, PSMB9, GCA, LCP2, F7, ITIH1, CFB, ACTN2, ITGAM, TFPI2*
**COAGULATION**	0.46	1.47	0.02	0.04	*C8A, MST1, TMPRSS6, MMP15, F3, PLEK, CTSE, MMP2, ITIH1, F13B, HPN, CFB, P2RY1, TFPI2, C1QA, F10, KLK8, LEFTY2, WDR1, GP9*
**ESTROGEN RESPONSE EARLY**	0.44	1.47	0.01	0.05	*PMAIP1, ESRP2, TJP3, RAPGEFL1, OVOL2, SFN, CLIC3, SLC19A2, SYT12, TOB1, SCNN1A, MAPT, ELF3, TFAP2C, CELSR2, ADCY1, PGR, TTC39A, MUC1, FOS*

ES- Enrichment Score; NES- Normalized Enrichment Score; NOM p-val- Nominal pvalue; FDR q-val- False discovery rate

Supplementary Table S4. Primers used in this study.

**RT-qPCR Primers**		
**Gene**	**Sequence**	**Direction**
Ankrd65	AGTGGCTAAGGGCATTGAAATA	Forward
Ankrd65	GGAGATGGCTGACAACTTCTAC	Reverse
Cbr2	GGTAGCCAGGGACATGATTAAC	Forward
Cbr2	GAGCTGTAGGTGATCAAGTTAGG	Reverse
Cdkn1a	TTAGGCAGCTCCAGTGGCAACC	Forward
Cdkn1a	ACCCCCACCACCACACACCATA	Reverse
Fermt1	CATGCAAATGGAGAGCAGCAG	Forward
Fermt1	TTCCCACCACAGAGCATAGTC	Reverse
Fermt3	ATGGAGGCTCAGGGAACAAA	Forward
Fermt3	CTTGGCCTTGAACTTTCGCT	Reverse
Gapdh	TCCATGACAACTTTGGCATTG	Forward
Gapdh	CAGTCTTCTGGGTGGCAGTGA	Reverse
Ddx4	GTGGAAATACTGGCAGAGCG	Forward
Ddx4	ATCCTGTTGAGCGTCTGACA	Reverse

**Genotyping primers**			
**Gene**	**Probe**	**Sequence**	**Direction**
Chek2	*Wildtype*	CTTGTCCTGCTGGACTCACA	Forward
Chek2	*Wildtype*	GCAACCGTTACCTACCCTGA	Reverse

Chek2	*Mutant*	CGGTCGCTACCATTACCAGT	Forward
Chek2	*Mutant*	CAGCGCTTATCCCAACACT	Reverse
Trp53	*Wildtype*	CAGCCTCTGTTCCACATACACT	Forward
Trp53	*Mutant*	AGGCTTAGAGGTGCAAGCTG	Forward
Trp53	*Common*	TGGATGGTGGTATACTCAGAGC	Reverse
Trp63	*Common*	ACCTGGCTTCCTTCTCATTG	Forward
Trp63	*Common*	CTTTGATACGCTGCTGCTTG	Reverse

Supplementary Data Files.

Supplementary Data 1: Bulk RNAseq_Ovary_Gene_Counts_ DEGs_groupwise
Supplementary Data 2: scRNAseq_Ovary_Clusters_Marker_Genes_DEGs_groupwise
Supplementary Data 3: scRNAseq_Oocyte_Subclusters_Marker_Genes_DEGs
